# Studying Human Fertility: Response to Slama et al. and Joffe et al.

**Published:** 2004-08

**Authors:** Joseph B. Stanford, David B. Dunson, Candace Tingen

**Affiliations:** Health Research Center, Department of Family and Preventive Medicine, University of Utah, Salt Lake City, Utah; National Institute of Environmental Health Sciences, National Institutes of Health, Department of Health and Human Services, Research Triangle Park, North Carolina, E-mail: dunson1@niehs.nih.gov

Slama et al. provide valuable comments on sampling frames to study fecundity, and we agree that the sampling frame is a major methodologic problem in fecundity studies of all designs. The current duration strategy of enrolling couples currently attempting pregnancy is a promising approach, particularly when couples are followed after enrollment to obtain detailed prospective information. Data from the menstrual cycles before enrollment can then be combined with detailed data from cycles during the study period using recently proposed statistical methods (Dunson 2003).

However, it is important to note that this innovative combination of retrospective and prospective designs still does not address the vexing problem of couples who do not have a clearly defined pregnancy attempt. Demographic surveys and qualitative research reveal that many—perhaps most—pregnancies are not exactly planned in the sense of an exactly defined onset of intention to become pregnant (Trussell et al. 1999). Even the onset of sexual intercourse without contraception may not always be easy to define reliably, with periods of use interspersed with periods of nonuse. Ultimately, a complete evaluation of this issue will need to include couples using contraception, at least at study enrollment. Some studies have done this, at least for barrier contraceptives (Eskenazi et al. 1995).

Joffe et al. comment on alternative retrospective designs that can be considered to address the problem of a nonrepresentative sample. We agree that prospective studies are limited by the fact that individuals willing to participate may not be representative of the general population (as in prospective epidemiologic studies of other heath outcomes). However, many of Joffe et al.’s comments on the prospective design are unduly negative. For example, the stated methodologic problem of it being “impossible to distinguish the approximately 3% of couples who are sterile from those who merely take a long time to conceive” is not specific to the prospective design, but a general issue in distinguishing sterility from infertility in the absence of known causes of sterility (Dunson et al. 2004).

The “best” design (if it exists) really depends on the scientific questions of interest. Retrospective and population-based studies have an important role in assessing population fecundability in demographic studies, in studying effective fecundability, and in surveillance for possibly significant environmental exposures. However, our focus is on studies investigating the potentially complex and time-varying effects of environmental exposures on biologic fecundability. Intercourse timing relative to ovulation has a critical role, not only in determining the overall probability of conception in a menstrual cycle, and hence time to pregnancy, but also in predicting later outcomes, such as early pregnancy loss (Wilcox et al. 1998). Confounding resulting from differences in exposed and unexposed individuals in their sexual behavior, including timing and frequency of intercourse, is a major concern. There can be problems even if the individuals have the same intercourse frequency because there is substantial variability in the timing of ovulation (Wilcox et al. 2000). In addition, prospective data on mucus and hormones potentially provide important information about biologic mechanisms.

For all of these reasons, we continue to recommend that whenever possible, detailed prospective data of the type that we have outlined should be collected in epidemiologic studies of fecundity, as well as in studies that seek to relate periconception exposures to later reproductive and developmental outcomes. Daily sampling of urine (via samples sent to the laboratory, or onsite with commercially available computerized devices) is one way to achieve this, but not the only one. We detailed other currently available and feasible approaches in our article (Tingen et al. 2004).
